# Similar Genetic Mechanisms Underlie the Parallel Evolution of Floral Phenotypes

**DOI:** 10.1371/journal.pone.0036033

**Published:** 2012-04-27

**Authors:** Wenheng Zhang, Elena M. Kramer, Charles C. Davis

**Affiliations:** Department of Organismic and Evolutionary Biology, Harvard University Herbaria, Cambridge, Massachusetts, United States of America; University of Lausanne, Switzerland

## Abstract

The repeated origin of similar phenotypes is invaluable for studying the underlying genetics of adaptive traits; molecular evidence, however, is lacking for most examples of such similarity. The floral morphology of neotropical Malpighiaceae is distinctive and highly conserved, especially with regard to symmetry, and is thought to result from specialization on oil-bee pollinators. We recently demonstrated that *CYCLOIDEA2*–like genes (*CYC2A* and *CYC2B*) are associated with the development of the stereotypical floral zygomorphy that is critical to this plant–pollinator mutualism. Here, we build on this developmental framework to characterize floral symmetry in three clades of Malpighiaceae that have independently lost their oil bee association and experienced parallel shifts in their floral morphology, especially in regard to symmetry. We show that in each case these species exhibit a loss of *CYC2B* function, and a strikingly similar shift in the expression of *CYC2A* that is coincident with their shift in floral symmetry. These results indicate that similar floral phenotypes in this large angiosperm clade have evolved via parallel genetic changes from an otherwise highly conserved developmental program.

## Introduction

Numerous comparative developmental genetic studies from diverse angiosperm lineages have shown that floral zygomorphy, or bilateral symmetry, has evolved via the repeated recruitment of *CYC2*-like genes of the TCP (*Teosinte Branched 1*, *CYCLOIDEA* and PCF) transcription factor family ([1], reviewed in [2], [3]). These studies have revealed frequent gene duplications during the evolution of *CYC2* [4], [5], [6], [7], [8], [9], [10], [11], [12], [13], [14] as well as a close correlation between the persistent expression of *CYC2* homologs in dorsal floral organs, especially the petals. While some of this data is from correlative patterns of gene expression from non–model species [8], [14], [15], [16], [17], [18], [19], [20], [21], [22], [23], it also includes zygomorphic model species with functional data [24], [25], [26], [27], [28], [29], [30]. Furthermore, once established in zygomorphic flowered lineages, modification of this *CYC2* program is associated with evolutionary variation in floral symmetry, including reversions to actinomorphy [15], [16], [17], [19], [20], [21], [22], [23], [31], [32]. This is particularly fascinating because floral zygomorphy has arisen at least 38 times [33], [34], [35] and is a hallmark feature of the most diverse angiosperm clades, including Asteraceae (24,000 sp.), Fabaceae (19,000 sp.), and Lamiales (23,000 sp.) [36], [37]. The evolution of floral zygomorphy is thus an important innovation in flowering plants and is thought to have arisen principally from specialization on insect pollinators [38], [39].

**Figure 1:**
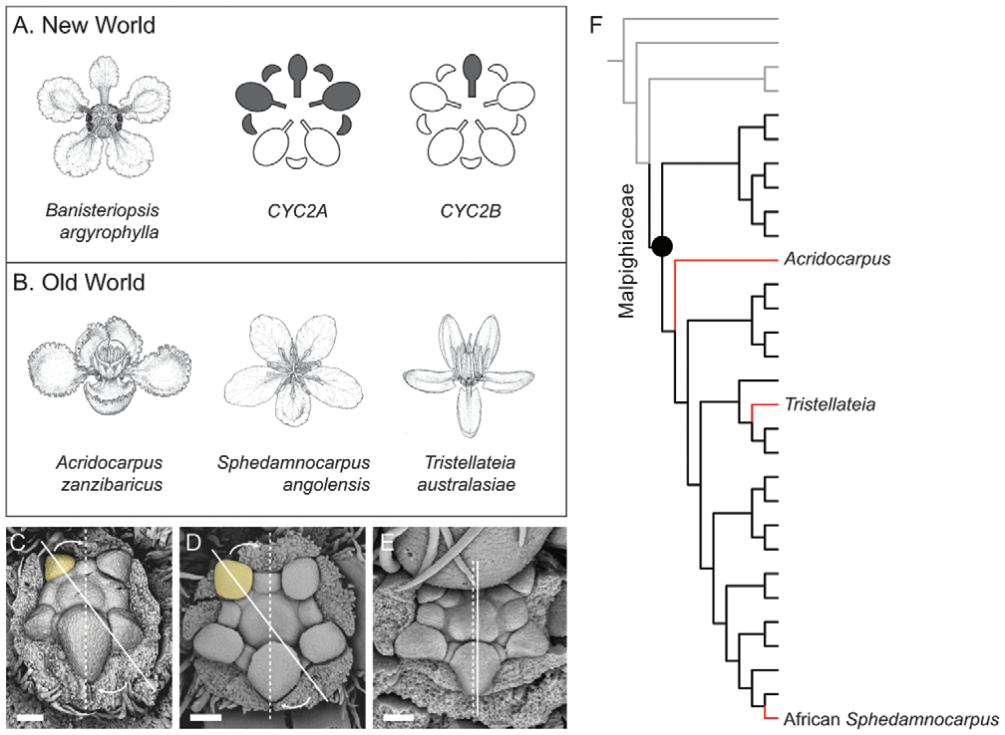
Floral morphology, development, and *CYC2* expression of Malpighiaceae. (A), *Banisteriopsis argyrophylla* illustrating the stereotypical New World floral morphology and pattern of *CYC2* expression in New World Malpighiaceae (expression shown in grey). (B), *Acridocarpus zanzibaricus*, *Sphedamnocarpus angolensis*, and *Tristellateia australasiae* (from left to right) represent three Old World floral phenotypes that have evolved in parallel from a similar New World-type ancestor. (C–E), Scanning electron micrographs showing the typical orientation of the two dorsal petals at the earliest stage of floral development in the New World Malpighiaceae species *Bunchosia glandulifera* (*C*) and *Heteropterys sp.* (*D*), and in the Old World species, *Tristellateia australasiae* (*E*). (F), Phylogeny depicting relationships of the three focal Old World clades: *Acridocarpus*, African *Sphedamnocarpus*, and *Tristellateia*. Grey lines highlight the radially symmetrical sister groups of Malpighiaceae, Centroplacaceae, and Elatinaceae [20]; black lines highlight Malpighiaceae species with the stereotypical New World floral morphology; red highlights the three Old World clades with parallel floral morphologies that have departed from the New World morphology. For reference, the banner petal of the New World Malpighiaceae is highlighted in yellow (*C* and *D*). Dotted lines = initial axis of floral symmetry; solid lines = final axis of floral symmetry; arrows indicate the shift in the axis of symmetry that takes place just before anthesis in New World Malpighiaceae [50]. Scale bars equal 100 μm.

The tropical plant clade Malpighiaceae exhibits a strong association between floral zygomorphy and specialist insect pollinators. The floral morphology of the more than 1,000 New World species of this clade is very distinctive and highly conserved, especially with regard to symmetry and pollinator reward [40], [41], [42]. The single upright/dorsal banner petal is strongly differentiated from other petals in the corolla whorl, and helps to orient and attract a limited suite of oil bee pollinators of the tribes Centridini, Tetrapedini, and Tapinotaspidini (Fig. 1*A*) [41], [42], [43], [44]. The banner petal in these New World species is therefore a critical component of this plant-pollinator mutualism [41]. In the mature flower, the very narrowed base of the petals provides the bees access to oil glands, which are borne in pairs on the abaxial surface of the sepals. This stereotypical floral morphology of New World Malpighiaceae, despite tremendous variation in vegetative and fruit morphology, appears to be due to their specialization on these oil-bee pollinators [42].

We recently established the likely genetic basis for this novel form of floral zygomorphy [20]. In this study, we identified two main lineages of *CYC2* in Malpighiaceae, *CYC2A* and *CYC2B*, which are derived from a duplication event coincident with the origin of the family. These loci are differentially expressed along the dorsoventral axis such that *CYC2A* is expressed in the dorsal banner petal and two adjacent lateral petals while *CYC2B* is restricted solely to the banner petal (Fig. 1*A*). This pattern of *CYC2* expression is conserved across three phylogenetically distant New World species, *Janusia guaranitica* A. Juss., *Byrsonima crassifolia* Kunth [20], and *Bunchosia glandulifera* (Jacq) H.B.K. (data from the latter species newly reported here, Fig. S1 *A*), that span the origin of the family and of this unique stereotyped floral morphology [20], [40]. In contrast, the radial flowered, species poor, outgroups of Malpighiaceae, Centroplacaceae and Elatinaceae, exhibit either no *CYC2* expression or broad radial *CYC2* expression at later stages of floral development, respectively [20]. A similar genetic and developmental transition, including CYC2 gene duplication and shift in the pattern of expression, was recently implicated in the transition from ancestrally radial flowers to derived bilateral flowers within the Dipsacales [23].

In contrast to high species diversity in the New World (∼1170 species in 59 genera), lineages of Malpighiaceae in the Old World are relatively species-poor (ca. 140 species in 14 genera) [45]. The Old World species were derived from seven independent migrations from the New World [40], [46], [47]. Importantly, these migrants have lost their specialist oil-bee pollinators, which do not occur in the Old World [48], [49], [50]. These clades also lack most of the characteristic floral features critical to the pollination syndrome of most New World Malpighiaceae and exhibit major architectural rearrangements of their floral morphology, having evolved either radially symmetrical flowers or shifted to a different kind of zygomorphy [40] (Fig. S2). Three Old World clades in particular–*Acridocarpus*, African *Sphedamnocarpus*, and *Tristellateia* (Fig. 1*B, F*)–have evolved strikingly similar floral morphologies in parallel and are highly diverged from their closest New World relatives. In each case the Old World flowers have maintained zygomorphic corollas, which they inherited from their New World ancestors (Fig. S2), but the plane of symmetry is dramatically reoriented such that they display two dorsal petals rather than a single conspicuous banner petal. In addition to these parallel changes in symmetry, each of the three clades has lost the oil glands entirely or shifted their contents to sugars, and *Acridocarpus* and *Tristellateia* have both evolved large poricidally dehiscent anthers, suggesting that these species are likely adapted to buzz pollination (Fig. 1*B*) [40], [45]. These changes in the Old World reflect shifts to new pollination mechanisms in which pollen or nectar, not oil, appears to be the principal pollinator reward [45], [51], [52].

Because *CYC2*-like genes likely play an important role in establishing floral symmetry in Malpighiaceae, we explored the possibility that similar modifications to this conserved *CYC2* symmetry program explain the parallel shifts in floral morphology that are observed in these three Old World clades. We show that in each case these species exhibit a loss of *CYC2B* function, and a strikingly similar shift in the expression of *CYC2A* that is coincident with their shift in floral symmetry. These results indicate that similar floral phenotypes in the Old World Malpighiaceae have likely evolved via parallel genetic changes from an otherwise highly conserved developmental program.

## Results and Discussion

### Floral development of the New and Old World Malpighiaceae

In order to understand the developmental basis of zygomorphy in both the New World and Old World Malpighiaceae, we analyzed the process by which their distinct floral forms initiate and mature. Our scanning electron micrographs demonstrate that the floral symmetry in New World species develops in the same manner as in most core eudicots [33], [53], [54]: the floral meristem is oriented with two dorsal petals (Fig. 1 *C* and *D*, Fig. S3 *A*, *B*, *D* to *I*). During development, however, this initial axis of symmetry is reoriented such that one of the two dorsal petals develops as the banner petal. This banner petal physically transitions to a dorsal medial position by rotation of the pedicel just before anthesis [50], thereby giving rise to the New World floral orientation. Establishing this secondary plane of floral symmetry is a key step in development of the New World floral zygomorphy because the placement of the banner petal in the dorsal medial position appears to be advantageous for orienting the oil bees [42], [50]. Thus, the stereotypical floral zygomorphy in New World Malpighiaceae appears to be characterized by the development of a novel axis of symmetry that is imposed onto the initial axis. Our earlier results demonstrated that the differential expression of *CYC2* genes is associated with this secondary plane of floral symmetry [20].

The three Old World clades that are the focus of our study here [*Acridocarpus*, African *Sphedamnocarpus*, and *Tristellateia* (Fig. 1*B*)] are each closely related to New World species that bear the typical banner petal floral morphology, but are oriented instead with two dorsal petals. From a developmental perspective, this pattern is not due to resupination of the flower [20] but rather to the maintenance of the incipient axis of symmetry without the subsequent reorientation that occurs in New World species (Fig. 1*E* and Fig. S3 *C*). In this regard, these Old World lineages exhibit a reversion to the ancestral floral orientation that characterizes rosids outside the Malpighiaceae [33], [53], [54].

### Independent functional loss of *CYC2B* in the Old World

**Figure 2:**
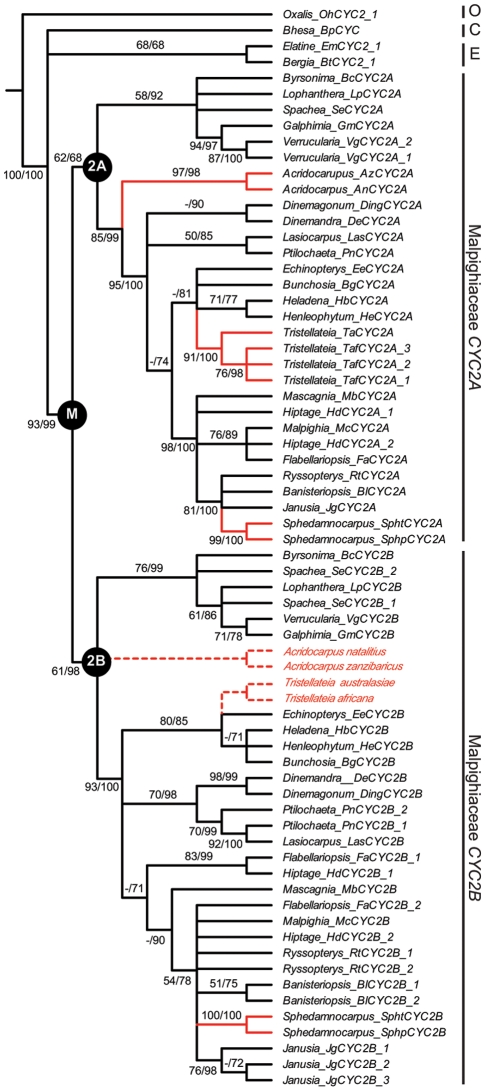
Phylogeny of *CYC2-*like genes for Malpighiaceae. Bayesian majority rule consensus topology shown; clades with >50% maximum likelihood (ML) bootstrap support and >60% Bayesian posterior probabilities depicted above lines, respectively. ML bootstrap support <50% indicated with a hyphen. Inferred gene tree is reflective of accepted species tree relationships [40]. Accessions highlighted in red include the three Old World clades examined here that exhibit parallel floral phenotypes–*Acridocarpus*, African *Sphedamnocarpus*, and *Tristellateia*. Accessions labeled with dotted lines signify inferred gene losses in *Acridocarpus natalitius*, *A. zanzibaricus*, *Tristellateia australasiae*, and *T. africana*. See Supplementary Table S2 for species identities and voucher information. C, Centroplacaceae; E, Elatinaceae; M, Malpighiaceae; O, Oxalidaceae.

As a first step to investigating *CYC2A* and *CYC2B* expression in these Old World lineages, we first identified *CYC2*-like homologs from *Acridocarpus* and *Sphedamnocarpus* using degenerate primers and exhaustive PCR clone screening. We did not detect *CYC2B* in two species of *Acridocarpus*, *A*. *natalitius* A.Juss. and *A. zanzibaricus* A.Juss. (Fig. 2), with confirmation by Southern analyses in *A*. *natalitius* (Fig. S4). This is consistent with our previous study of another Old World species, *Tristellateia australasiae* A. Rich., which has similarly lost *CYC2B* [20]. In contrast, the two species of *Sphedamnocarpus*, *S. pruriens* Szyszył. and *S. transvaalicus* Burtt Davy, maintain both copies of *CYC2A* and *CYC2B* (Fig. 2). Locus-specific reverse transcription (RT)-PCR, however, reveals that the *CYC2B* copy is not expressed in *S. pruriens* at the late stages of floral development (Fig. S1 *C*). These findings are in sharp contrast to those in New World Malpighiaceae, which possess and express both *CYC2A* and *CYC2B* [20] (also Fig. S1 *A*). In all three Old World clades that have been sampled, the banner petal paralog *CYC2B* has been lost or is not expressed, demonstrating a striking correspondence between the loss of *CYC2B* function and the loss of the New World dorsal banner petal morphology. One potentially interesting hypothesis that emerges from these results relates to the divergence time estimates of these three Old World clades and the observed pattern of *CYC2B* loss. *Acridocarpus* and *Tristellateia* diverged from their closest New World relatives during the Eocene (∼55 million years ago [mya]) and Oligocene (∼30 mya), respectively [46], [47]. In contrast, *Sphedamnocarpus* diverged from its closest New World relatives much more recently, during the Miocene (∼20 mya) [46], [47]. This raises the possibility that there has not been sufficient time for *CYC2B* to have been lost from *Sphedamnocarpus*, hence its presence in the genome despite its lack of expression.

### Independent shifts of *CYC2A* expression pattern in the Old World

**Figure 3:**
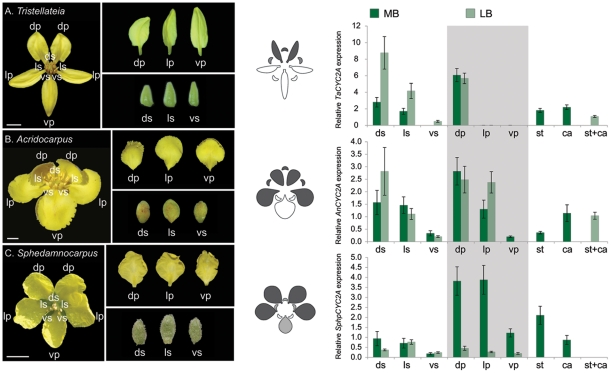
Quantitative RT-PCR (qRT-PCR) expression of *CYC2-*like genes for the parallel floral morphologies in the Old World Malpighiaceae *Tristellateia australasiae* (A) *Acridocarpus natalitius* (B) and *Sphedamnocarpus pruriens* (C). Grayscale shading on floral diagrams summarizes the relative strength of the spatial pattern of *CYC2* expression in the corolla and calyx. qRT-PCR expression data was determined for dissected floral organs at mid and late stages. Expression levels are relative to the control *β-tubulin*. Error bars represent standard errors. ds, dorsal sepal; ls, lateral sepal; vs, ventral sepal; dp, dorsal petal; lp, lateral petal; vp, ventral petal; st, stamens; ca, carpels; MB, medium buds ∼40–60% of full size buds; LB, large buds ∼70–90% of full size buds. Scale bars equal 5 mm.

**Figure 4:**
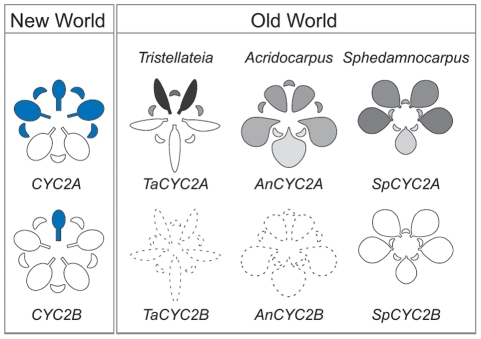
Summary of *CYC2*-like gene expression. Expression of *CYC2*-like genes in New World Malpighiaceae [20] and in three parallel shifts in the Old World Malpighiaceae clades represented, from left to right, by *Tristellateia australasiae*, *Acridocarpus natalitius*, and *Sphedamnocarpus pruriens.* The blue shading of the New World Species indicates late stage *CYC2* gene expression [20] (Fig. S1 *A*). The gradient shading in Old World species, from white to black, indicates increasing intensity of *CYC2* expression, respectively.

To examine how *CYC2A* expression has been modified in these Old World clades, we investigated its expression pattern using quantitative RT–PCR, which revealed that expression in the dorsal region of the corolla is significantly greater than in the ventral region for all species (Fig. 3 and statistics Table S1). The expression in the dorsal region of the calyx is also significantly greater than in the ventral region for *T. australasiae* and *A. natalitius* (Fig. 3 and statistics Table S1). These observations demonstrate that the three parallel shifts in floral symmetry in these Old World Malpighiaceae share very similar changes in the pattern of *CYC2A* expression. Interestingly, there are differences in the details in each of these cases: *Tristellateia* maintains *CYC2A* in only the two dorsal petals while *Acridocarpus CYC2A* expression is expanded to include the lateral petals; and *Sphedamnocarpus CYC2A* is even more broadly expressed, but only very weakly in the ventral petal (Fig. 3 and summary Fig. 4). In *T. australasiae* and *A. natalitius* (Fig. 3 *A* and *B*), the spatial expression of *CYC2A* is maintained in the dorsal region during development. In contrast, *CYC2A* expression in *S. pruriens* (Fig. 3*C*) is significantly decreased in all petals during the latest developmental stages. These distinctions underscore the fact that each of these three lineages independently transitioned to their Old World morphology in response to the loss of their specialist New World oil bee pollinators. *CYC2A* is also expressed in the stamens and carpels, but at relatively low levels of gene activity in all three species (Fig. 3). Moreover, the configuration of stamens relative to the petals in these Old World species is identical to their closest zygomorphic New World relatives. Low levels of *CYC2* expression suggests that *CYC2* may not play a role in the development of the androecium and gynoecium in these species of Malpighiaceae [20] (Fig. 3).

### The genetic basis of parallel floral phenotypes in Old World Malpighiaceae

Taken together, our current and previous [20] findings in the Malpighiaceae suggest a genetic model for both the evolution of zygomorphy in the family and its subsequent modification as lineages dispersed to novel environments involving a new pollinator selective regime. This model is critical to the ecological interactions with the oil bee pollinators and, accordingly, is conserved in diverse lineages that maintain this mutualism. *CYC2* expression, particularly the banner petal expression of *CYC2B*, correlates with the secondary axis of floral symmetry that reorients New World flowers to place the single banner petal in the dorsal medial position. In multiple separate instances, however, members of the family have migrated to the Old World where they have lost their oil bee pollinators as well as their critical banner petal zygomorphy. In the three cases examined here, the shift in pollinators is always associated with a loss of floral reorientation and functionality of the *CYC2B* locus, in addition to a novel axis of symmetry in *CYC2A* expression. A similar decay in the zygomorphic program in response to a change in pollination syndrome has been demonstrated for a single clade of Veroniceae [21], but our sampling provides strong evidence in the context of multiple parallel events within a narrowly circumscribed plant clade. In addition, the examples here represent parallel, pollinator-mediated, modifications of the ancestral program to yield a new pattern of zygomorphy, rather than a reversion to radial symmetry similar to what is found in the close relatives of Malpighiaceae. These contrasting New and Old World patterns reinforce the conclusion that *CYC2* homologs are critical to floral symmetry in this diverse family. Our current efforts are focused on examining earlier stages to determine how the dynamics of *CYC2* expression correlate with the developmental shift in the plane of floral symmetry we have characterized in New World Malpighiaceae. Finally, these findings reinforce the observations that the *CYC2* module is consistently recruited for the evolution of zygomorphy in angiosperms [2], [3], and newly demonstrates that once this developmental module is established within a large clade it can be modified by strikingly similar parallel genetic changes.

## Materials and Methods

### Specimen collections

Specimens of *Acridocarpus natalitius* are from the National Botanical Garden, Lowveld, South Africa; *A. zanzibaricus* from Dar es Salaam, Tanzania; *Sphedamnocarpus pruriens* from Pretoria, South Africa; *Bunchosia glandulifera*, *Byrsonima lucida* DC., and *Heteropterys sp.* from the Kampong Tropical Botanical Garden, the Montgomery Botanical Center in Florida, and the University of California at Davis, USA; and *Tristellateia australasiae* and *Galphimia gracilis* Bartl. are from cultivated plants at Harvard University in Massachusetts, USA (see Table S2).

### Ethics statement

Collections from Tanzania and South Africa were sent as part of a collaboration with Frank M. Mbago (Curator, Herbarium DSM, Botany Department, University of Dar es Salaam) and Robert H. Archer (Researcher, National Herbarium PRE, South African National Biodiversity Institute), respectively, who held the necessary permission to collect in their respective countries.

### Isolation of *CYC2A* and *CYC2B*

To isolate *CYC2*-like genes from our target species we used degenerate primers designed for Malpighiaceae and their closest relatives (Table S3). These included 11 degenerate forward primers and seven degenerate reverse primers. We exhaustively screened our target species using 77 primer pair combinations to identify the best primer pair for screening *CYC2* gene copies. The optimal *CYC2* primer pair (Forward: 5′-GCIMGIAARTTYTTYGAYYTKCAA; Reverse: 5′-GCYCKYGCYCTIGCYYTHKCYCTWGA) was chosen based on its ability to amplify many major clades of Malpighiales, including especially the sister families of Malpighiaceae. *CYC2*-like amplicons spanning the TCP and R domains were obtained following our previous methods [20]. More than 200 clones were screened for this effort.

### Sequence alignments and phylogenetic analyses

The newly acquired sequences of *CYC2*-like genes from *Acridocarpus natalitius*, *A. zanzibaricus*, *Sphedamnocarpus pruriens*, and *S. transvaalicus* were aligned with a previously available matrix including several ingroup accessions of Malpighiaceae, and the outgroup families, Elatinaceae, Centroplacaceae, and Oxalidaceae, by eye with reference to the translated amino acid sequences using MacClade 4.06 [55]. We applied the WAG+G model of amino acid evolution to the aligned *CYC2* data set as determined by the AIC criterion in ProtTEST [56]. One thousand maximum likelihood bootstrap replicates were conducted using RAxML-VI-HPC [57]. Bayesian analyses were implemented in MrBayes ver. 3.1.2 [58] under the same optimal model using default priors for the rate matrix, branch lengths, and gamma shape parameter. A Dirichlet distribution was used for the base frequency parameters and an uninformative prior was used for the starting tree topology. Four chains were initiated with a random starting tree and run for two million generations sampled every 1,000 generations. Stationarity was determined using Tracer v1.4.1. (http://tree.bio.ed.ac.uk/software/tracer/). We sampled from the posterior distribution to calculate clade posterior probabilities following a burn-in of 1,000 trees. All DNA sequences of the newly acquired *CYC2*-like genes have been deposited in GenBank, under accession numbers JQ723742 through JQ723749.

### Southern hybridization

Ten µg of genomic DNA was digested from *Acridocarpus natalitius* with restriction enzymes (i.e., *Hin*dIII, *Eco*RI, and *Hin*dIII plus *Eco*RI), fractionated on 0.8% agarose gels, and blotted onto a positively charged nylon membrane (GE Healthcare Bio-Sciences Corp., Piscataway, NJ) following the protocol in Zhang *et al*. [20]. A fragment containing the 3' end of the TCP domain and the variable region between the TCP and R domains was used as a template to synthesize probes for detecting *CYC2*-like genes. A mixture of *CYC2A* sequences (*AnCYC2A* of *A. natalitius* and *BcCYC2A* of *Byrsonima crassifolia* [20]) and *CYC2B* sequences (*BcCYC2B* of *B. crassifolia* [20]) in equal molar concentration was used as a template to synthesize our ^32^P labeled probe. We previously showed that the number of bands in the *Eco*RI digest is a reliable indicator of *CYC2* copy number [20]. Here, we identified a single band in the *Eco*RI digest (Fig. S4 *A*). In the *Hin*dIII single digest and *Hin*dIII+*Eco*RI double digest, we expected more than one band due to the presence of a single restriction site of *Hind*III within the probed region (Fig. S4 *B*). As before [20], this result is identical to our results from PCR and cloning. These results further demonstrate that our PCR/clone screens provide the same estimate of gene copy number as our low stringency Southern hybridizations.

### RNA sample preparations

We examined two developmental stages for organ specific *CYC2* expression. Floral organs from the latest stages were dissected in the field from multiple flower buds ranging in size from ∼70–90% of bud size just before anthesis. Earlier stage flower buds were also collected from each species. All materials were preserved in cryogenic containers, and were processed in the lab using the RNAqueous kit (Ambion, Austin, TX, USA). Floral organs from earlier, medium stage samples, ∼50% of flower bud size just before anthesis, were dissected in the lab from a single bud. These buds were dissected using the RNA*later*®-ICE Kit (Ambion-Applied Biosystems, Austin, TX, USA). Frozen buds were transferred to 1 ml of –80°C RNA*later*®-ICE. Vacuum infiltration was applied followed by incubation in the same solution at –20°C for 16 hours. Floral dissection was then performed using a dissecting microscope at room temperature. The micro-dissected samples were processed using the RNAqueous Micro kit (Ambion, Austin, TX, USA). DNA contamination was removed with a DNA-free kit (Ambion, Austin, TX, USA). RNA quality was assessed using the Agilent 2100 Bioanalyzer with the RNA 6000 Nano Labchip® kit for our pooled samples and the RNA 6000 Pico Labchip kit for each organ dissected from a single bud (Agilent Technologies, Palo Alto, CA, USA). Additionally, RNA quality in all five petals and sepals were analyzed separately for *Acridocarpus natalitius*, *Sphedamnocarpus pruriens,* and *Tristellateia australasiae*.

### Reverse transcription (RT)-PCR

RT-PCR was performed as previously described [20] using locus specific primers (Table S4) to examine the expression of *CYC2*. The sequence identity of RT-PCR fragments was further confirmed by sequencing.

### Quantitative RT-PCR and statistical analysis

qRT-PCR reactions were conducted using PerfeCTa® SYBR® Green FastMix®, Low ROX™ (Quanta BioSciences, Inc., Gathersburg, MD) using the Stratagene Mx3005P QPCR System. Class I *β-tubulin* was used as a control to normalize the qRT-PCR [59]. The stable expression of *β-tubulin* was confirmed by semi-quantitative RT-PCR (data not shown). *CYC2* expression levels were calculated relative to *β-tubulin* using the 2^-ΔΔCT^ method [60]. Absence of genomic DNA was confirmed with our *β-tubulin* control, which spanned a ∼90-bp intron region. No *β-tubulin* amplicons were observed for the higher molecular weight intron bearing copy. Thus, our RNA preparations were free of genomic contamination. The identity of all amplicons was confirmed by sequencing. One biological replicate (i.e., one extraction from >30 flower buds from an individual plant) was analyzed for the latest stages; three biological replicates (i.e., three extractions from three flower buds from an individual plant) were analyzed for the medium sized bud stages. Three technical replicates (i.e., three separate qRT-PCRs from a single extracted sample) were analyzed for each biological replicate. Standard errors were calculated from all technical replicates. The statistical significance of the differential pattern of spatial gene expression for the medium sized bud samples was examined for the sepals and petals, respectively, as implemented in the software package REST© 2009 (Technische UniversitŠt München, Qiagen) [61]. We tested the null hypothesis that there was no significant difference between the spatial pattern of gene expression within the calyx and corolla whorls (e.g., relative expression levels in the ventral versus the dorsal petals). Our non-parametric analysis included 10,000 random reallocations of the relative spatial expression data for each pair-wise comparison we made (e.g., ventral petal expression versus dorsal petal expression). For example, to determine whether RNA abundance of *CYC2A* in the dorsal petals is significantly higher than that in the ventral petal we applied REST to normalize the *CYC2* expression ratios of *beta-Tubulin*, correct the fold changes based on primer efficiencies, and calculate p-values through a pair-wise reallocation randomization analysis (using 10,000 replicates) of the two groups (e.g., ventral petal expression versus dorsal petal expression) [61]. These results are reported in Table S1.

### Morphology-based character state reconstruction of floral symmetry

We used maximum likelihood (ML) character state reconstruction as implemented in Mesquite version 2.6 [62] to infer the evolution of floral symmetry in Malpighiaceae and its closest relatives, Elatinaceae and Centroplacaceae. The analysis was done using the phylogeny and methods described by Zhang et al. (2010), and by scoring each species as zygomorphic or radial flowered.

### Scanning electronic microscopy

Inflorescences of *Bunchosia glandulifera*, *Byrsonima lucida, Galphimia gracilis, Heteropterys sp*., and *Tristellateia australasiae* were fixed in FAA in the field and transferred to 70% ethanol for storage. Young inflorescences from each species were prepared in 2% osmium for 4 hours at room temperature, washed, and dehydrated in a graded series of ethanol. Samples were then coated with Platinum-Palladium and observed with a Zeiss EVO 50 microscope at 10–20 keV. Images were enhanced with Adobe Photoshop.

## Supporting Information

Figure S1 **Locus-specific RT-PCR for** ***CYC2*** **-like gene expression in Malpighiaceae.** (A), *Bunchosia glandulifera* shows the conserved *CYC2A* and *CYC2B* expression in New World Malpighiaceae [20]. (B–C), The temporal pattern of *CYC2* expression in the Old World Malpighiaceae *Acridocarpus zanzibaricus* (B) and *Sphedamnocarpus pruriens* (C). *ACTIN*-specific primers were used as a positive control. Abbreviations are as follows: dp, dorsal petal; lp, lateral petal; vp, ventral petal; s+c, stamens and carpals; uc, upper calyx; lc, lower calyx; MB, medium buds ∼40–60% of full size buds (MB_1_, ∼40–50%; MB_2_, ∼50–60%); LB, large buds ∼70–90% of full size buds; FL, open flowers. Scale bars equal 5 mm.
(TIF)

Figure S2 **Ancestral character state reconstruction of floral symmetry.** Maximum likelihood analysis indicates the relative likelihood of floral symmetry at each node. Accessions highlighted in red include the three Old World clades examined here that exhibit parallel floral phenotypes–*Acridocarpus,* African *Sphedamnocarpus*, and *Tristellateia*.
(TIF)

Figure S3 **Floral development of Malpighiaceae.** (A–I), All Malpighiaceae species, *Bunchosia glandulifera* (A), *Heteropterys sp.* (B), *Tristellateia australasiae* (C), *Byrsonima lucida* (D–F), and *Galphimia gracilis* (G–I), have an initial axis of floral symmetry with two petals in the dorsal position relative to the axis. In New World Malpighiaceae this initial axis (F, I; dotted line) is replaced by a final axis of floral symmetry (F, I; solid line) in which the single banner petal (in yellow) is in the dorsal-most position. The Old World species [e.g., *Tristellateia australasiae* (C)] do not exhibit this secondary reorientation. Asterisks = the inflorescence apices; dotted lines = initial axis of floral symmetry; solid lines = final axis of floral symmetry; arrows indicate the rotation of the floral axis achieved before anthesis [50]. Note, the direction of reorientation varies from flower-to-flower and can be predicted using the position of the carpel primordia and the inner-most, banner petal [20]. Scale bars equal 400 μm in (A–E, G–H), and 100 μm in (F, I).
(TIF)

Figure S4 ***CYC2*** **Southern hybridization results for** ***Acridocarpus natalitius*** **.** (A), Restriction digests using *Eco*RI (E), *Hin*dIII (H), and *Eco*RI+*Hin*dIII (E+H) are shown for genomic DNA of *A. natalitius*. Lane contains *CYC2* plasmid DNA as controls to test probe efficiency. (B), Restriction cut site was determined from sequence analysis and are indicated on the *CYC2* gene copy shown at bottom. Arrows and numbers indicate molecular size markers (in base pairs). The number of bands in the *Eco*RI digest reflects the *CYC2* copy number based on our previous study [20]. The single band in the *Eco*RI digest suggests one copy of the *CYC2* gene in *Acridocarpus natalitius*. In the *Hin*dIII and double digests, we expected more than one band due to the presence of a restriction site within the probed region.
(TIF)

Table S1 **Statistical strength of differential pattern of spatial gene expression within the corolla and calyx whorls.**
(DOC)

Table S2 **Species sampled, with collection locations, voucher information, and** ***CYC2*** **loci.**
(DOC)

Table S3 **Degenerate PCR primers used in this study.**
(DOC)

Table S4 **qRT-PCR annealing temperatures, amplification efficiencies, and primer sequences used in this study.**
(DOC)
