# Supplementary figures and images for: Similar Genetic Mechanisms Underlie the Parallel Evolution of Floral Phenotypes
Source: PLoS One. 2012 Apr 27;7(4):e36033. doi: 10.1371/journal.pone.0036033 (PMC3338646; doi:10.1371/journal.pone.0036033)

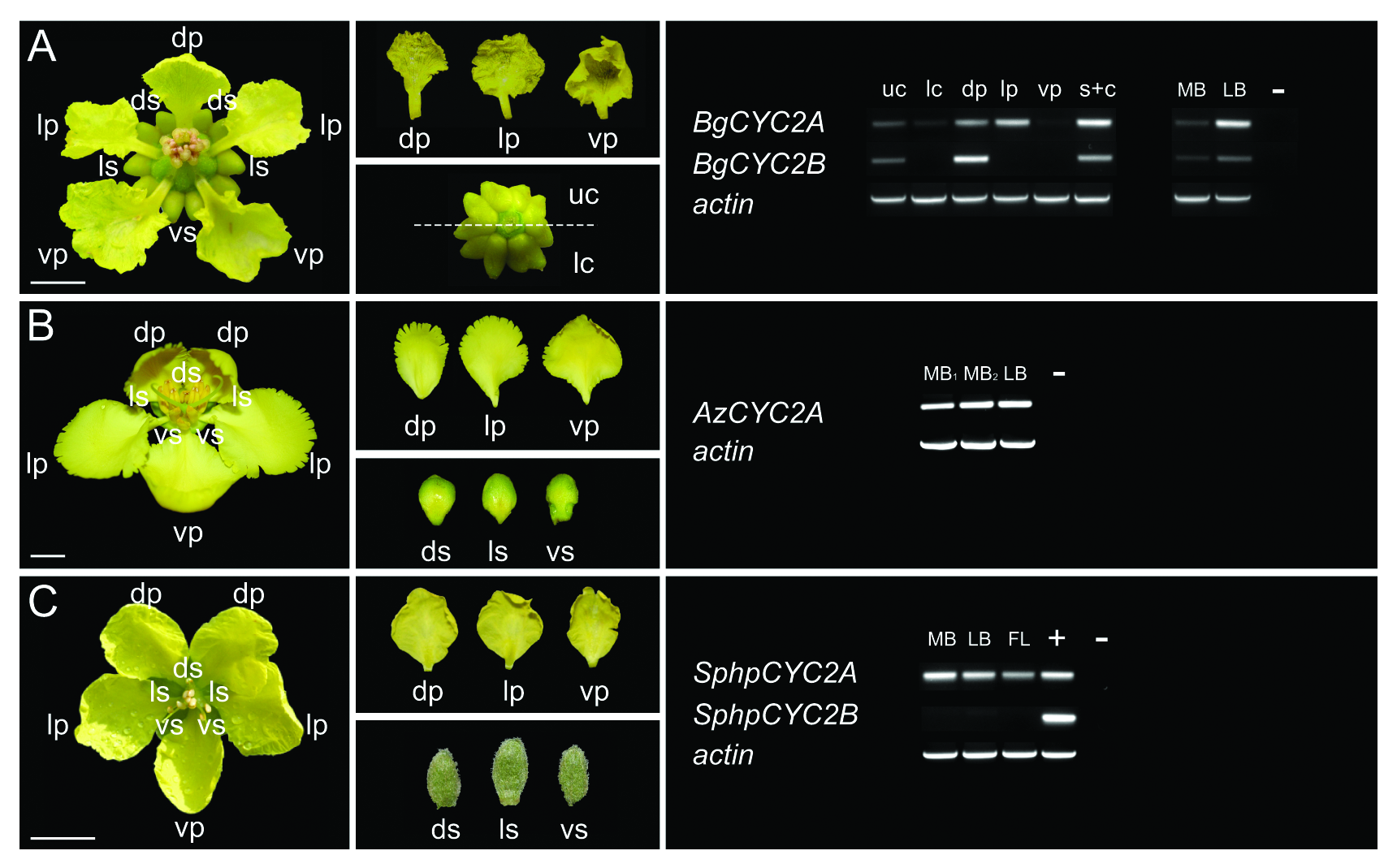

Supplement: Figure S1 — Locus-specific RT-PCR for CYC2 -like gene expression in Malpighiaceae. (A), Bunchosia glandulifera shows the conserved CYC2A and CYC2B expression in New World Malpighiaceae [20]. (B–C), The temporal pattern of CYC2 expression in the Old World Malpighiaceae Acridocarpus zanzibaricus (B) and Sphedamnocarpus pruriens (C). ACTIN-specific primers were used as a positive control. Abbreviations are as follows: dp, dorsal petal; lp, lateral petal; vp, ventral petal; s+c, stamens and carpals; uc, upper calyx; lc, lower calyx; MB, medium buds ∼40–60% of full size buds (MB1, ∼40–50%; MB2, ∼50–60%); LB, large buds ∼70–90% of full size buds; FL, open flowers. Scale bars equal 5 mm. (TIF) [file pone.0036033.s001.tif]

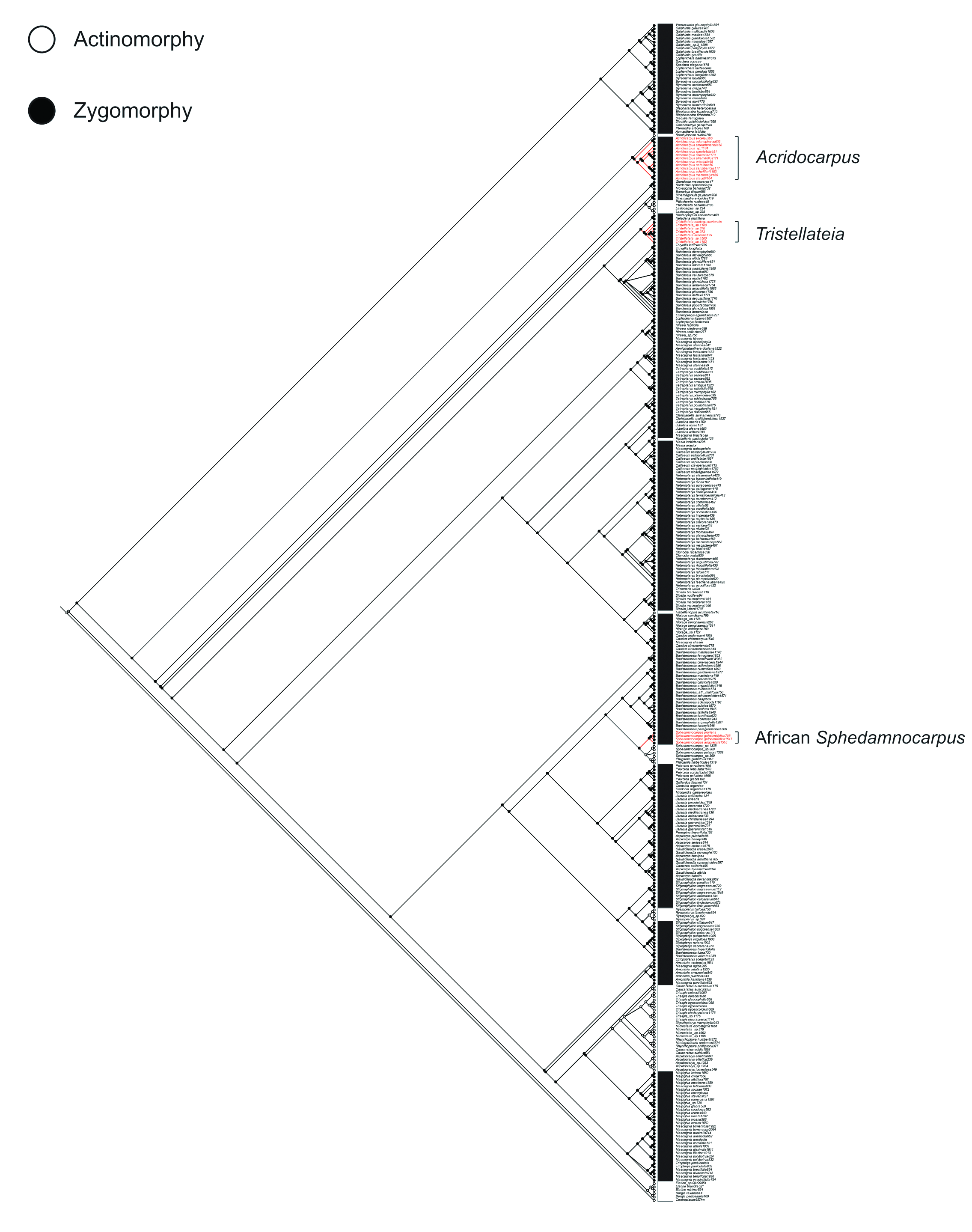

Supplement: Figure S2 — Ancestral character state reconstruction of floral symmetry. Maximum likelihood analysis indicates the relative likelihood of floral symmetry at each node. Accessions highlighted in red include the three Old World clades examined here that exhibit parallel floral phenotypes–Acridocarpus, African Sphedamnocarpus, and Tristellateia. (TIF) [file pone.0036033.s002.tif]

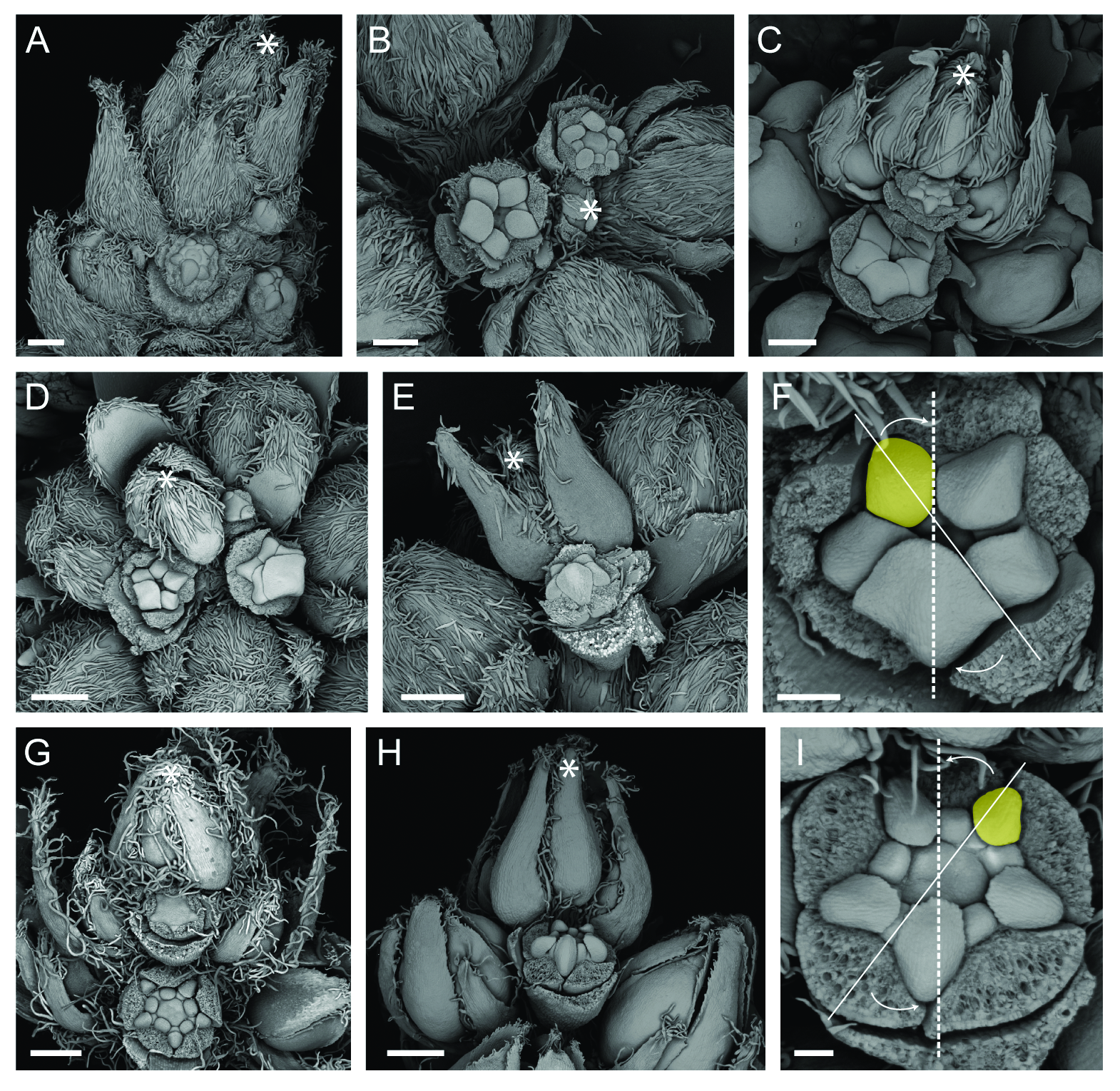

Supplement: Figure S3 — Floral development of Malpighiaceae. (A–I), All Malpighiaceae species, Bunchosia glandulifera (A), Heteropterys sp. (B), Tristellateia australasiae (C), Byrsonima lucida (D–F), and Galphimia gracilis (G–I), have an initial axis of floral symmetry with two petals in the dorsal position relative to the axis. In New World Malpighiaceae this initial axis (F, I; dotted line) is replaced by a final axis of floral symmetry (F, I; solid line) in which the single banner petal (in yellow) is in the dorsal-most position. The Old World species [e.g., Tristellateia australasiae (C)] do not exhibit this secondary reorientation. Asterisks = the inflorescence apices; dotted lines = initial axis of floral symmetry; solid lines = final axis of floral symmetry; arrows indicate the rotation of the floral axis achieved before anthesis [50]. Note, the direction of reorientation varies from flower-to-flower and can be predicted using the position of the carpel primordia and the inner-most, banner petal [20]. Scale bars equal 400 μm in (A–E, G–H), and 100 μm in (F, I). (TIF) [file pone.0036033.s003.tif]

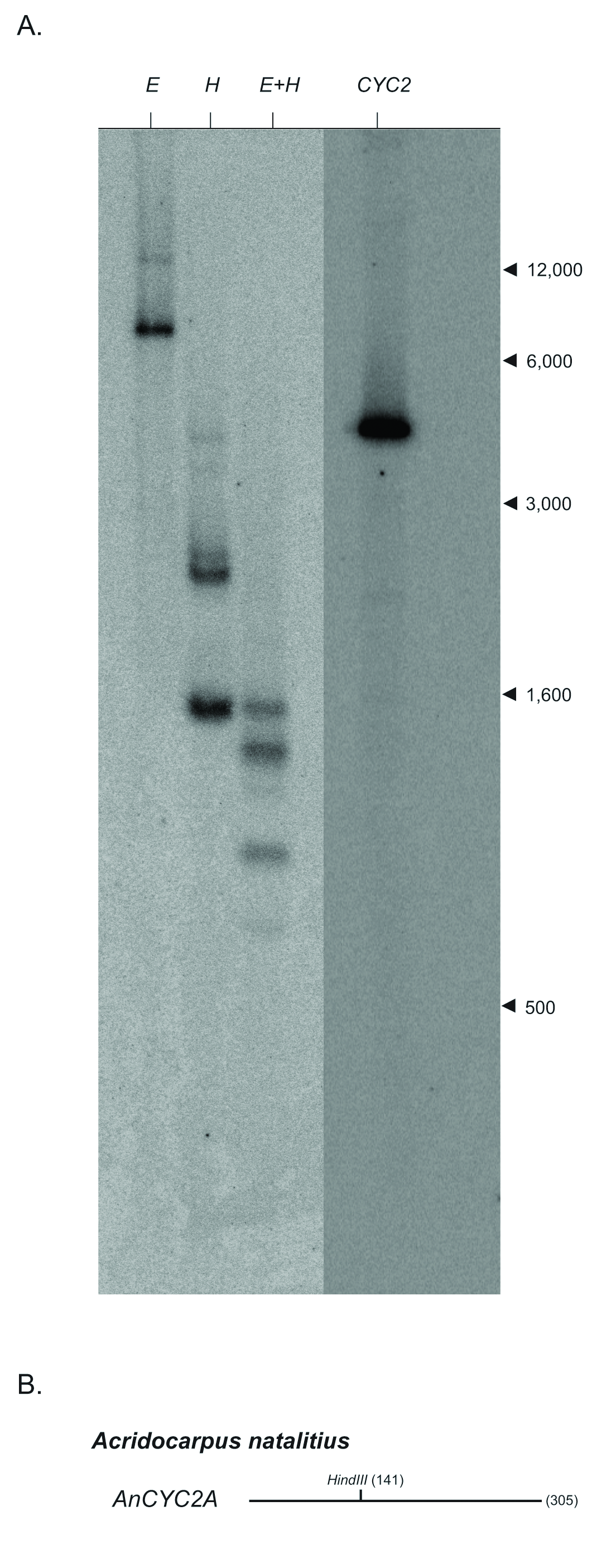

Supplement: Figure S4 — CYC2 Southern hybridization results for Acridocarpus natalitius . (A), Restriction digests using EcoRI (E), HindIII (H), and EcoRI+HindIII (E+H) are shown for genomic DNA of A. natalitius. Lane contains CYC2 plasmid DNA as controls to test probe efficiency. (B), Restriction cut site was determined from sequence analysis and are indicated on the CYC2 gene copy shown at bottom. Arrows and numbers indicate molecular size markers (in base pairs). The number of bands in the EcoRI digest reflects the CYC2 copy number based on our previous study [20]. The single band in the EcoRI digest suggests one copy of the CYC2 gene in Acridocarpus natalitius. In the HindIII and double digests, we expected more than one band due to the presence of a restriction site within the probed region. (TIF) [file pone.0036033.s004.tif]
