# Supplementary material for: Similar Genetic Mechanisms Underlie the Parallel Evolution of Floral Phenotypes
Source: PLoS One. 2012 Apr 27;7(4):e36033. doi: 10.1371/journal.pone.0036033 (PMC3338646; doi:10.1371/journal.pone.0036033)
Supplement: Table S1 — Statistical strength of differential pattern of spatial gene expression within the corolla and calyx whorls. (DOC) [file pone.0036033.s005.doc]

**Table S1. Statistical strength of differential pattern of spatial gene expression within the corolla and calyx whorls.**

| Pairwise comparisons | *Tristellateia australasiae* | *Acridocarpus natalitius* | *Sphedamnocarpus pruriens* |
| --- | --- | --- | --- |
| dp vs vp | ** | ** | * |
| lp vs vp | - | - | ** |
| dp vs lp | ** | - | - |
| ds vs vs | * | * | - |
| ls vs vs | ** | ** | - |
| ds vs ls | - | - | - |

*, significance P<0.05; **, significance P<0.01; -, non-significant. dp, dorsal petal; lp, lateral petal; vp, ventral petal; ds, dorsal sepal; ls, lateral sepal; vs, ventral sepal.
