# Supplementary material for: Similar Genetic Mechanisms Underlie the Parallel Evolution of Floral Phenotypes
Source: PLoS One. 2012 Apr 27;7(4):e36033. doi: 10.1371/journal.pone.0036033 (PMC3338646; doi:10.1371/journal.pone.0036033)
Supplement: Table S2 — Species sampled, with collection locations, voucher information, and CYC2 loci. (DOC) [file pone.0036033.s006.doc]

**Table S2. Species sampled, with collection locations, voucher information, and *CYC2* loci.**

| Species | Location | Voucher | *CYC2* loci | |
| --- | --- | --- | --- | --- |
|  |  |  | 2A | 2B |
| *Acridocarpus natalitius* A.Juss. | Cult. Lowveld National Botanical Garden, Nelspruit, South Africa | Zhang, Archer & Boufford 150 (A) | *AnCYC2A* | - |
| *Acridocarpus zanzibaricus* A.Juss. | Dar es Salaam, Tanzania | Zhang, Mbago & Boufford 154 (A) | *AzCYC2A* | - |
| *Bunchosia glandulifera* (Jacq.) H.B.K. | Cult. OEB, Harvard U. | Zhang 168 (MICH) | *BgCYC2A* | *BgCYC2B* |
| *Sphedamnocarpus pruriens* Szyszył. | Pretoria, South Africa | Zhang, Archer & Boufford 148 (A) | *SphpCYC2A* | *SphpCYC2B* |
| *Sphedamnocarpus transvaalicus* Burtt Davy | Pretoria, South Africa | Zhang, Archer & Boufford 152 (A) | *SphtCYC2A* | *SphtCYC2B* |

Note. A = Arnold Herbarium, Harvard University Herbaria; MICH = University of Michigan Herbarium.
