# Supplementary material for: Similar Genetic Mechanisms Underlie the Parallel Evolution of Floral Phenotypes
Source: PLoS One. 2012 Apr 27;7(4):e36033. doi: 10.1371/journal.pone.0036033 (PMC3338646; doi:10.1371/journal.pone.0036033)
Supplement: Table S3 — Degenerate PCR primers used in this study. (DOC) [file pone.0036033.s007.doc]

**Table S3. Degenerate PCR primers used in this study.**

| Name | 5’ to 3’ | Forward / Reverse |
| --- | --- | --- |
| cycF2b | GCIMGIAARTTYTTYGAYYTKCAAGAYATG | F |
| cycF2c | GCGAGMAARTTYTTYGAYYTGCAAGAYATG | F |
| cycF2d | GCIMGIAARTTYTTYGAYYTKCAA | F |
| cycF2e | GCGAGMAARTTYTTYGAYYTGCAA | F |
| cycF3a | AARTTYTTYGAYYTKCAAGAYATGYTRGGK | F |
| cycF3b | AARTTYTTYGAYYTGCAAGAYATGYTAGGK | F |
| cycF4a | GAYYTGCAAGAIATKYTIGRKTTY | F |
| cycF4b | GAYYTGCAAGAYATGYTRGGKTTT | F |
| cycF5a | ATGYTIGRKTTYGAYARRGCIAGYAAH | F |
| cycF5b | ATGYTAGGKTTYGAYARRGCIAGYAAA | F |
| cycF5c | ATGYTAGGKTTYGAYARRGCIAGYAAC | F |
| cycR3a | TCYCTIGCYCTIGCYCTIGCYTT | R |
| cycR3b | TCYCTWGCYCMYGCYCTHGCYYT | R |
| cycR3c | TCTCTTGCYCKTGCYCTHGCTTT | R |
| cycR1a | GCYCKYGCYCTIGCYYTHKCYCTWGA | R |
| cycR1b | GCYCTTGCYCTYGCTYTWGCCCTAGA | R |
| cycR2a | TCYCTWGCYCKYGCYCTIGCYYTHKCYCT | R |
| cycR2b | TCTCTTGCYCTTGCYCTHGCTYTWGCCCT | R |
