# Supplementary material for: Similar Genetic Mechanisms Underlie the Parallel Evolution of Floral Phenotypes
Source: PLoS One. 2012 Apr 27;7(4):e36033. doi: 10.1371/journal.pone.0036033 (PMC3338646; doi:10.1371/journal.pone.0036033)
Supplement: Table S4 — qRT-PCR annealing temperatures, amplification efficiencies, and primer sequences used in this study. (DOC) [file pone.0036033.s008.doc]

**Table S4. qRT-PCR annealing temperatures, amplification efficiencies, and primer sequences used in this study.**

| Gene name | Taxon name | Forward primer (5’ to 3’) | Reverse primer (5’ to 3’) | Amplicon size (bp) | Annealing temperature (°C) | Amplification efficiency (%) |
| --- | --- | --- | --- | --- | --- | --- |
| *AnCYC2A* | *Acridocarpus natalitius* | GTTAGGGTTTGACAGGGCAAG | TGCTTAGCAAGAACTGCGATT | 249 | 60 | 98 |
| *AnTUB1* | *Acridocarpus natalitius* | AAGATTAGAGAGGAGTACCCTGATAGA | GCAAGTGACACCACTCATTGTC | 255 | 60 | 110 |
| *AzCYC2A* | *Acridocarpus zanzibaricus* | GTTAGGGTTTGACAGGGCAAG | TGCTTAGCAAGAACTGCGATT | 249 | - | - |
| *BgCYC2A* | *Bunchosia glandulifera* | CCCTTGAATGGCTTCTTCTT | GCTTAGCAAAAAGTGGGATTTT | 210 | - | - |
| *BgCYC2B* | *Bunchosia glandulifera* | GACCTTTTGGGGTTTGATAGAG | TTCAATCTTCTTTCACTGGATTTG | 242 | - | - |
| *SphpCYC2A* | *Sphedamnocarpus pruriens* | CCCTTGAATGGCTTCTTCTC | TTGCTTACAAACAAGTGGGATT | 239 | 60 | 105 |
| *SphpCYC2B* | *Sphedamnocarpus pruriens* | AAAACCCTTGAGTGGCTTCTTAC | TTCCTTGACAAGAACCAAAGC | 243 | - | - |
| *SphpTUB1* | *Sphedamnocarpus pruriens* | TCAGGGAGGAGTACCCTGATAGA | GCAAGTGACACCACTCATTGTC | 251 | 60 | 86 |
| *TaCYC2A* | *Tristellateia australasiae* | TTAGGGTTTGACAGGGCAAG | GCTTAGCAAGAAGTGGGATTT | 247 | 60 | 81 |
| *TaTUB1* | *Tristellateia australasiae* | TCAGGGAGGAGTACCCTGATAGA | CAAGTGACGCCACTCATTGTT | 250 | 60 | 98 |

Note: RT-PCR for *Bunchosia glandulifera* and *Acridocarpus zanzibaricus* were conducted in this study following the protocol described in reference [20]; bp = base pair.
